# The burden of musculoskeletal disorders in the Middle East and North Africa (MENA) region: a longitudinal analysis from the global burden of disease dataset 1990—2019

**DOI:** 10.1186/s12891-023-06556-x

**Published:** 2023-05-31

**Authors:** Yazan A. Al-Ajlouni, Omar Al Ta’ani, Rand Mushasha, Justin Lin Lee, Jaishree Capoor, Mitul R. Kapadia, Ruth Alejandro

**Affiliations:** 1 New York Medical College School of Medicine, Valhalla, NY 10595 USA; 2 University of Pittsburgh Medical Center, Pittsburgh, PA 15260 USA; 3 Charité – Universitätsmedizin Berlin Charitéplatz 1, 10117 Berlin, Germany; 4 One Brooklyn Health, Brooklyn, NY 11212 USA; 5 Department of Pediatrics and Orthopedics, University of California, San Francisco, USA

**Keywords:** Burden of disease, Musculoskeletal disorders, MENA region, Risk factors, DALYs

## Abstract

**Background:**

Musculoskeletal (MSK) disorders are one of the main causes of disability among adults globally. The burden of MSK disorders varies greatly between different regions and is the highest in low- and middle income- countries. This study sought to investigate trends in the burden of MSK disorders across the MENA region, utilizing the GBD 2019 dataset.

**Methods:**

This ecological study utilized data from the Global Burden of Disease (GBD) to report on the burden of musculoskeletal (MSK) disorders in The Middle East and North Africa (MENA) region between 1990 and 2019. Our analysis involved descriptive statistics and sociodemographic trends and did not employ any specific statistical analyses. Using age-standardized rates of prevalence and disability-adjusted life-years (DALYs), we reported trends in the burden of MSK disorders, as well as national variation between different countries. Furthermore, we analyzed trends in risk factors contributing to MSK disorders by age and gender.

**Results:**

The longitudinal analysis from 1990 to 2019 showed an increase in the age-standardized rate for prevalence and DALYs of MSK disorders by 5% and 4.80%, respectively. Low back pain continued to be the most prevalent MSK condition, while RA and other MSK disorders had the largest percentage increase for DALYs between 1990 and 2019. The study found that Afghanistan had the lowest age standardized DALYs rate attributed to MSK disorders, while Iran, Turkey, and Jordan had the highest. Further, Syria showed the most dramatic decrease while Saudi Arabia had the most notable increase in age standardized DALY rates from 1990 to 2019. In 2019, occupational risks, high body mass index, and tobacco smoking were the main risk factors for MSK disorders, with occupational risks being the largest contributor, and between 1990 and 2019, there was a decrease in the contribution of occupational risks but an increase in the contribution of high body mass index as a risk factor.

**Conclusion:**

This study highlights the significant burden of MSK disorders in the MENA region, with various risk factors contributing to its increasing prevalence in recent decades. Further research is needed to better understand the underlying factors and potential interventions that could improve health outcomes. Addressing MSK disorders should be a public health priority in the region, and efforts should be made to develop effective strategies to prevent and manage this debilitating condition.

## Introduction

Among adults, Musculoskeletal (MSK) disorders are one of the main causes of disability [1–3]. Overall, and according to global epidemiological data, there are five common conditions constituting MSK disorders, including rheumatoid arthritis (RA) [4], osteoarthritis (OA) [5], low-back pain (LBP) [6], neck pain (NP) [7, 8] and gout [5]. Any other conditions that fit the definition of MSK disorders (e.g., any type of discomfort to irreversible and disabling injury affecting the muscles, joints, tendons, bones, cartilage, ligaments and nerves) are combined into a category titled “other MSK disorders” [9]. According to the Global Burden of Disease (GBD) study, low back pain was the leading cause of years lived with disability in most countries and territories, and musculoskeletal conditions as a group were a main driver of noncommunicable disease (NCD)-related disability burden [10].

In an earlier iteration of the GBD study in 2010, MSK disorders were shown to have the fourth greatest burden on the health of the world’s population [1, 10, 11] It is estimated that approximately 6.7% of the global disability-adjusted life years are attributed to MSK disorders [10, 11]. When measured in years lived with disability, 21% of global disability is attributed to MSK conditions [8, 12]. In terms of prevalence, neck pain was found to be the most prevalent MSK disorder globally, affecting around 15% of the population in 2019. Osteoarthritis was the second most prevalent MSK disorder, affecting around 4% of the population in 2019. Additionally, MSK disorders were found to be responsible for a significant proportion of years of life lost (YLLs) due to premature mortality, particularly for rheumatoid arthritis, osteoarthritis, and low back pain [13–15]. Moreover, the burden of MSK disorders varies considerably by region and country, with neck pain being most prevalent globally and varying from 6% in sub-Saharan Africa to over 20% in Western Europe and North America [4, 8]. The prevalence of osteoarthritis is particularly high in high-income countries, whereas rheumatoid arthritis is more common in middle-income countries. Of note, the burden of disability due to MSK conditions is projected to increase in future years, especially in low- and middle-income countries [4, 8]. This is due to population growth, aging, and increased behavioral and work-related risk factors (e.g., obesity, injury, and a sedentary lifestyle) [9, 16].

Despite this large burden globally, epidemiological studies aiming to better understand the impact of MSK on different populations continue to be very limited within the literature. Mainly, this can be due to MSK disorders being perceived to be rarely fatal and mostly irreversible [17], thus shifting epidemiological and clinical research priorities to other more pressing disorders. Previous research has reported that the burden of MSK disorders varies greatly among different countries, especially over the last decade. More specifically, the burden tends to be higher in low- and middle- income countries [18, 19], where resource allocation is mainly focused on other health conditions among the population that may be deemed more urgent and fatal. Furthermore, MSK disorders severely limit people’s activities and restrict their participation in society by working less, retiring early, increasing their need for social support and disability pensions. Hence, understanding and addressing the burden of MSK disorders is of increased importance in low- and middle- income countries to facilitate people’s ability to contribute to the development of local society and economy.

Over the past few decades, The Middle East and North Africa (MENA) region became increasingly affected by political conflicts, social unrest, as well as displacement and immigration. Within this context, already fragile health systems and data were further weakened by the resulting protracted wars, forced displacement, and economic decline [20]. To date, the MENA region has lacked population-based studies describing the prevalence, burden, and risk factors for MSK disorders. Additionally, and unlike developed countries, rehabilitation medicine is not a well-established medical specialty in the region, which further exacerbates the burden of MSK disorders and deficiency of data. In the MENA region, the literature on this topic has been limited to studies focusing on the burden of other health outcomes, studies utilizing survey-based measures among a small sample of the population, or studies based on patient-populations [21, 22]. To better understand the burden of MSK disorders and evaluate whether health policies should focus on addressing them among the population in the MENA region, there is a strong call to quantify the burden of MSK disorders and understand the risk factors that contribute to them among this highly diverse population.

Upon this basis, this study sought to investigate trends in the burden of MSK disorders across the MENA region, utilizing the GBD 2019 dataset. The findings of this study will provide, *to the very best of our knowledge,* the very first comprehensive estimate of the burden of MSK disorders among a very understudied population in health research. Such findings can be essential to our understanding of the urgency to address musculoskeletal disorders in the MENA region and have potential to direct intervention measures and policy making efforts.

## Methods

### Data

This study used the 2019 version of the GBD dataset [23], which provides estimates for multiple measures such as deaths, incidence, prevalence, years of life lost (YLLs), years lived with disability (YLDs), and disability-adjusted life-years (DALYs) due to a total of 369 diseases and injuries across 87 different risk factors, for both sexes in 204 different countries and territories [8, 24]. Data is updated annually, allowing for comparison over time from 1990 to 2019 across all the measures mentioned.

The GBD study is conducted by the Institute for Health Metrics and Evaluation (IHME) at the University of Washington in Seattle, with financial support from various institutions, including the World Bank, the National Institutes of Health, and the Bill & Melinda Gates Foundation. Notably, the Bill & Melinda Gates Foundation provided substantial funding to the IHME, making it a significant sponsor of the project [8, 25].

Detailed data, including numbers and age-standardized rates (ASRs) of prevalence and DALYs by cause, sex, age, and location, were downloaded from the Global Health Data Exchange website. DALYs were calculated as the sum of YLLs, based on a reference maximum observed life expectancy, and YLDs based on standardized disability weights for each health state. The DALY rate is a better descriptor of noncommunicable chronic diseases due to their consequences of low mortality and high disability rates.

### GBD data analysis

The GBD study draws data from multiple sources such as vital registration systems, censuses, sample registration systems, surveys, medical facilities, and death certificates. The data is processed, adjusted for covariates, and modeled using standardized tools: Cause of Death Ensemble model (CODEm), spatiotemporal Gaussian process regression (ST-GPR), and DisMod-MR. The GBD Compare website allows for downloading and interactive visualization of results.

The GBD study team implements various approaches to address missing data, which vary depending on the nature of the missingness. For data that is considered missing at random, they utilize multiple imputation techniques, whereas data that is not missing at random may be addressed using inverse probability weighting or other similar methods. Additionally, the team prioritizes maintaining the quality and consistency of the data and documents their methods thoroughly to ensure transparency in their handling of missing data during the analysis process [26, 27].

Further details about processing and modeling strategies can be found in the literature [4, 5, 8, 24, 25, 28–32].

### Measures

We report trends in rates of prevalence and DALYs during 1990–2019, for population aged ≥ 5 years old. We further explored attributable risk factors and decomposed the changing trend in DALYs to assess underlying causes. We used R software package to graphically represent our findings [33].

#### DALYs

The burden of a particular disability is measured using DALYs, which is a common metric [34]. This metric calculates the total number of YLLs and the number of YLDs. To calculate DALYs, YLLs are added to YLDs, where YLLs are based on a reference maximum life expectancy and YLDs are based on standardized disability weights for each health condition [24].

#### Prevalenc*e*

Prevalence is a measure of the percentage of people in a population who have a specific disease or condition at a given time. This metric is often used in epidemiology to determine the burden of a disease within a population.

#### Musculoskeletal disorders

We utilized the American College of Rheumatology and the International Statistical Classification of Diseases and Related Health Problems (ICD-10) to define Musculoskeletal Disorders as referenced in the GBD dataset. The main musculoskeletal disorders in the GBD dataset are 1) low back pain; 2) occupationally related low back pain; 3) neck pain; 4) OA; 5) rheumatoid arthritis (RA); 6) gout; 7) low bone mineral density and 8) other MSK conditions. According to the ICD-10 categorization, other MSK conditions as reported in the GBD dataset include 13 other diagnoses, such as infectious arthropathies, inflammatory polyarthritis, and deforming dorsopathies.

#### Risk factors

A detailed description of the GBD 2019 methods for estimating the burden of disease associated with risk factors has been published elsewhere [24, 29]. For the purposes of this study, all risk factors contributing to MSK disorders as part of the GBD 2019 dataset were included: 1) tobacco use (defined as current daily or occasional use of any smoked tobacco product); 2) occupational risks (including occupational injuries; ergonomic factors; and occupational exposure to particulate matter, fumes and glasses, carcinogens, noise, and asthmagens); 3) kidney dysfunction (defined as estimated glomerular filtration rate (eGFR) less than 60 ml/min/1·73m2 or albumin to creatinine ratio (ACR) greater than or equal to 30 mg/g); 4) high body-mass index (defined as BMI greater than 25 kg/m2). Risk factor exposures were estimated by using population-representative survey and surveillance data and geospatial Gaussian process regression models that borrowed strength across time and geography.

#### MENA region

We used the GBD dataset categorization of MENA countries, which included 21 countries listed as follows: Afghanistan, Algeria, Bahrain, Egypt, Iran, Iraq, Jordan, Kuwait, Lebanon, Libya, Morocco, Oman, Palestine, Qatar, Saudi Arabia, Sudan, Syria, Tunisia, Turkey, United Arab Emirates, Yemen.

## Results

### Burden of MSK disorders at the regional and national levels

**Fig. 1:**
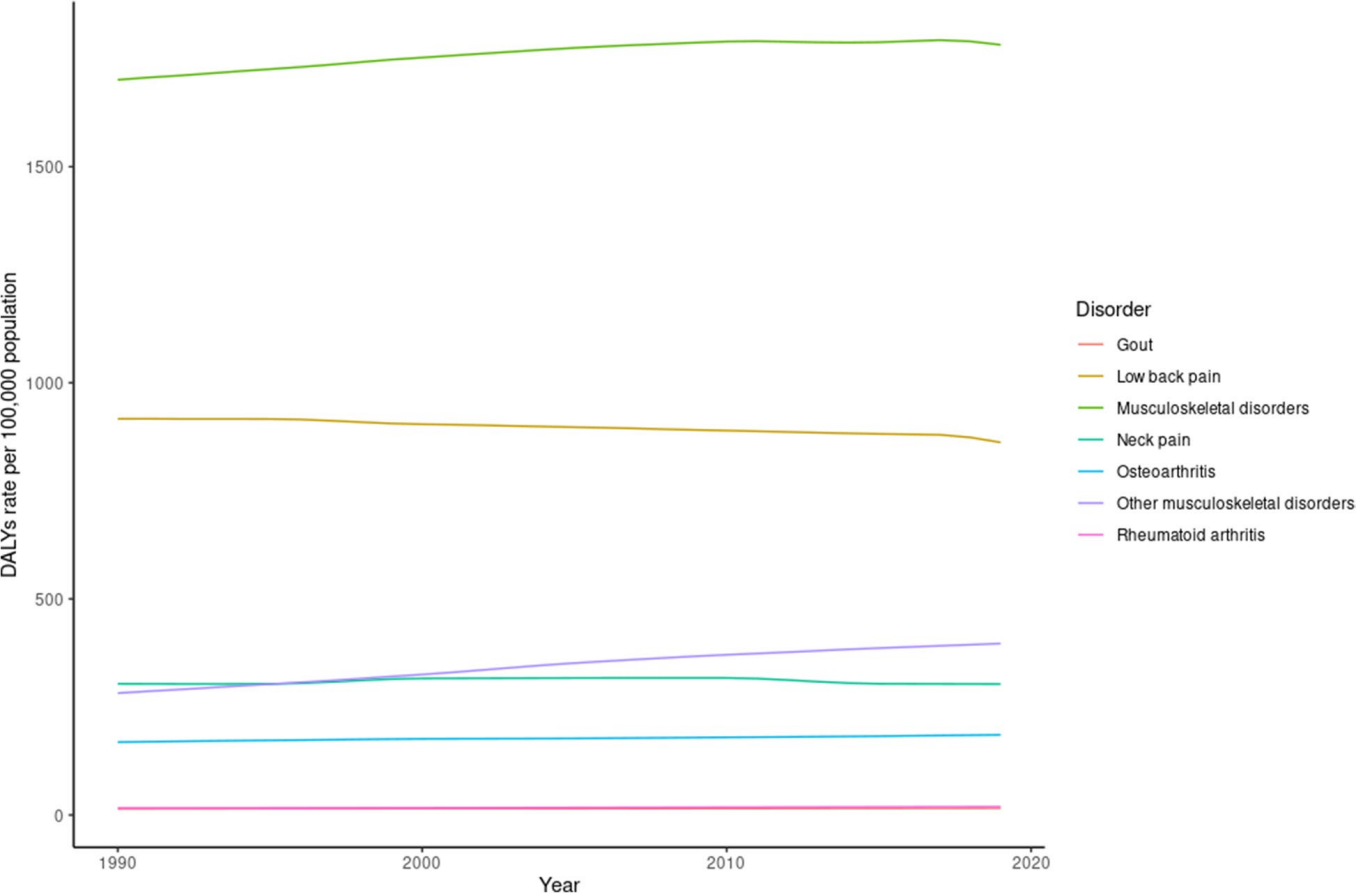
DALYs rate per 100,000 population for different MSK disorders between 1990 and 2019

**Fig. 2:**
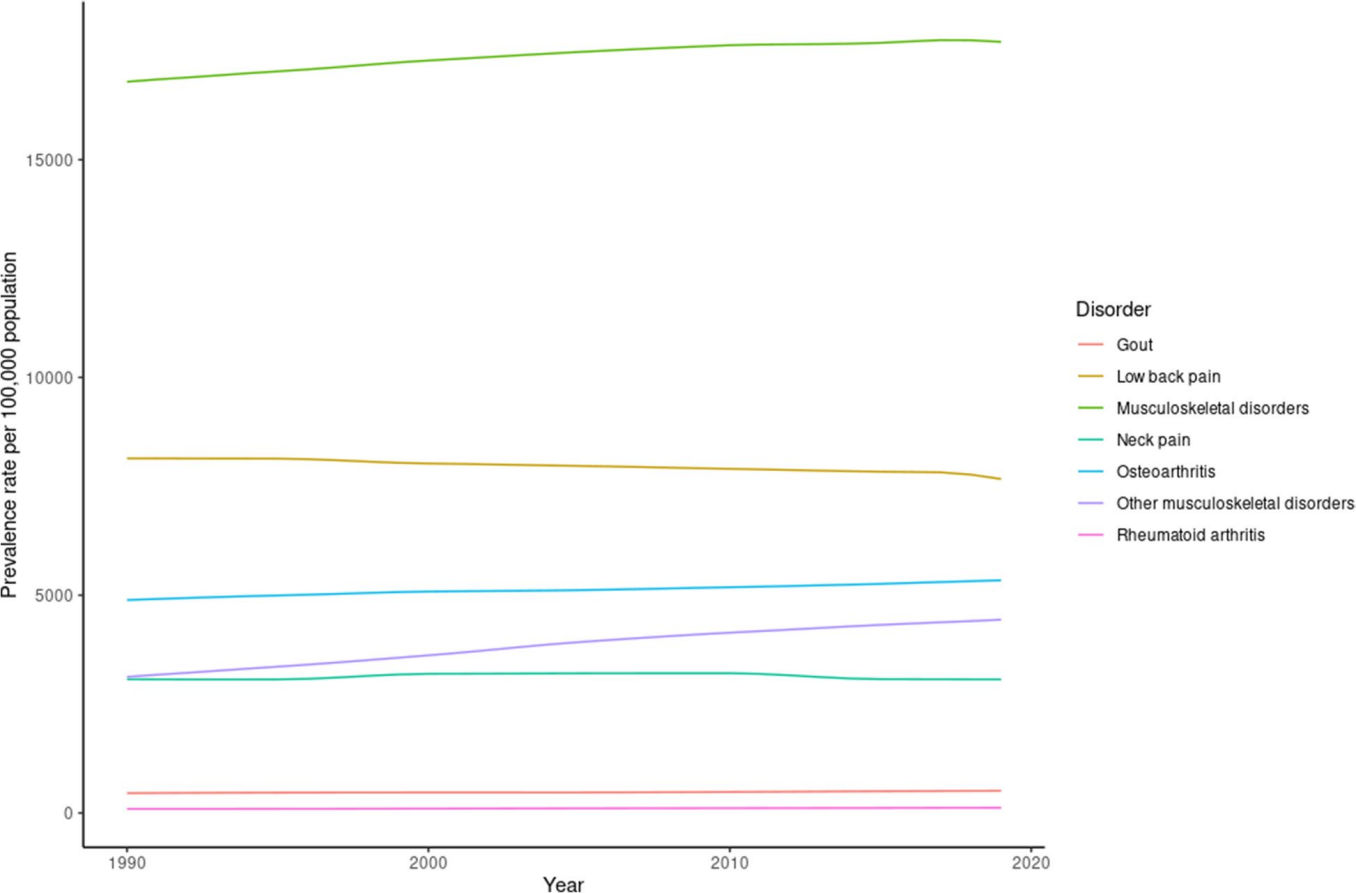
Prevalence rate per 100,000 population for different MSK disorders between 1990 and 2019

Tables 1 and 2 show the all age-prevalence and DALY numbers, rates, and percentage changes for different subcategories of MSK disorders in 1990 and 2019. In 2019, the age-standardized rate prevalence and DALYs for all MSK disorders among both genders was 17706 [95% UI = 16641, 18794] and 1782 [95% UI = 1278, 2366], respectively. Between 1990 and 2019, the age-standardized rate for prevalence and DALYs increased by 5% and 4.80%, respectively. Low back pain continued to be the most prevalent MSK condition in 2019, exhibiting the highest contribution to age-standardized prevalence rates (52.2%), followed by Osteoarthritis (22.6%), other MSK disorders (19.2%), and neck pain (18.4%). Relatively similar trends were observed when measuring the burden of disease, where low back pain was the highest contributor to DALYs in 2019 (56.5%), followed by neck pain (17.6%), other MSK disorders (16.8%), and osteoarthritis (7.4%). Between 1990 – 2019, the largest percentage increase for DALYs was witnessed for RA and other MSK disorders, with 18.80% and 41% increase, respectively. On the other hand, despite contributing the most to the burden of MSK disorders, lower back pain was the only condition that decreased in burden (-6%) between 1990 and 2019. These trends are shown in Figs. 1 and 2.

**Table 1:** All age-prevalence number, rate, and percentage change for different subcategories of MSK disorders in 1990 and 2019

**Subcategory**		**All-age prevalence number 1990**	**All-age prevalence number 2019**	**Change (%)**	**Age-standardized rate 1990**	**Age-standardized rate 2019**	**change (%)**
**All MSK**	Males	18215667 (16871641, 19564455)	45978773 (42660461, 49207290)	152	15554 (14567, 16558)	16201 (15182, 17192)	4
	Females	20263166 (18858550, 21659205)	50904285 (47504990, 54356782)	151	18073 (16951, 19211)	19329 (18169, 20539)	7
	Both	38478834 (35773264, 41249804)	96883058 (90259212, 103000000)	152	16790 (15749, 17796)	17706 (16641, 18794)	5
**Rheumatoid arthritis**	Males	59824 (50543, 70357)	187234 (160224, 218812)	213	51 (44, 59)	65 (56, 76)	27
	Females	160303 (139816, 184633)	485593 (427971, 552315)	202	139 (123, 157)	180 (161, 202)	29
	Both	220127 (190013, 253220)	672827 (588698, 767999)	206	94 (83, 107)	121 (107, 136)	29
**Osteoarthritis**	Males	3943144 (3522566, 4404960)	11332552 (10154001, 12663002)	187	4372 (3933, 4843)	4786 (4300, 5304)	9
	Females	4739201 (4253663, 5300776)	13272059 (11904574, 14783765)	180	5417 (4879, 6018)	5925 (5326, 6569)	9
	Both	8682345 (7793117, 9681655)	24604611 (22080960, 27327135)	183	4889 (4405, 5422)	5343 (4816, 5908)	9
**Gout**	Males	663236 (527304, 825932)	1904810 (1510650, 2359399)	187	694 (552, 863)	766 (609, 955)	10
	Females	193846 (151909, 243165)	550791 (428960, 689886)	184	212 (167, 266)	239 (188, 300)	13
	Both	857082 (681502, 1072547)	2455601 (1940521, 3045022)	187	455 (363, 569)	509 (406, 634)	12
**Low back pain**	Males	10747984 (9445777, 11965233)	22962287 (20057295, 25957224)	114	8517 (7534, 9495)	7791 (6885, 8801)	-9
	Females	9329928 (8180686, 10549570)	20276752 (17798803, 22840437)	117	7735 (6848, 8721)	7529 (6674, 8452)	-3
	Both	20077912 (17626717, 22491145)	43239039 (37773370, 48819120)	115	8141 (7213, 9121)	7668 (6798, 8636)	-6
**Neck pain**	Males	2810447 (2221464, 3608854)	6980885 (5471254, 9029997)	148	2369 (1872, 3017)	2368 (1870, 3009)	0
	Females	4285541 (3368243, 5536,322)	10399283 (8102644, 13411805)	143	3802 (2963, 4871)	3825 (2983, 4905)	1
	Both	7095988 (5588333, 9132,517)	17380168 (13535872, 22315483)	145	3069 (2410, 3897)	3067 (2408, 3894)	0
**Others**	Males	2501949 (1928040, 3142588)	9745055 (7919637, 11892814)	290	2058 (1597, 2574)	3230 (2612, 3910)	57
	Females	4895916 (4095183, 5767293)	15803242 (13373233, 18546231)	223	4242 (3564, 4991)	5743 (4896, 6723)	36
	Both	7397865 (6003352, 8901651)	25548297 (21263742, 30311500)	245	3126 (2570, 3753)	4438 (3735, 5262)	42

**Table 2:** All age-DALYs number, rate, and percentage change for different subcategories of MSK disorders in 1990 and 2019

**Subcategory**		**All-age DALYs number 1990**	**All-age DALYs number 2019**	**change (%)**	**Age-standardized rate 1990**	**Age-standardized rate 2019**	**change (%)**
**All MSK**	Males	1915710 (1361775, 2548391)	4691517 (3332516, 6209809)	145	1577 (1128, 2093)	1610 (1143, 2140)	2
	Females	2106744 (1519566, 2792477)	5268392 (3776569, 6949231)	150	1829 (1317, 2415)	1967 (1421, 2605)	8
	Both	4022454 (2874294, 5339466)	9959909 (7136729, 13201313)	148	1701 (1218, 2254)	1782 (1278, 2366)	5
**Rheumatoid Arthritis**	Males	10558 (7504, 13892)	29765 (21154, 39782)	182	9 (7, 12)	11 (8, 14)	22
	Females	25925 (18461, 33883)	73808 (52917, 98218)	185	23 (17, 30)	28 (20, 37)	22
	Both	36483 (26371, 47677)	103572 (74242, 136655)	184	16 (12, 21)	19 (14, 25)	19
**Osteoarthritis**	Males	135769 (67728, 271453)	391163 (193904, 775910)	188	150 (75, 301)	165 (83, 329)	10
	Females	163850 (82346, 324468)	461728 (232257, 914017)	182	187 (94, 370)	207 (104, 406)	11
	Both	299619 (150145, 599219)	852891 (425290, 1687138)	185	169 (84, 337)	185 (93, 370)	10
**Gout**	Males	21020 (13086, 30264)	60224 (38226, 86615)	187	22 (14, 31)	24 (15, 34)	9
	Females	6117 (3840, 8872)	17290 (10859, 24790)	183	7 (4, 10)	7 (5, 11)	0
	Both	27137 (16907, 38843)	77514 (48808, 111730)	186	14 (9, 20)	16 (10, 23)	14
**Low back pain**	Males	1231428 (857096, 1661479)	2630930 (1819851, 3521727)	114	970 (684, 1301)	885 (618, 1186)	-9
	Females	1042837 (733799, 1389231)	2268370 (1589905, 3026890)	118	860 (607, 1146)	836 (593, 1117)	-3
	Both	2274265 (1590072, 3033870)	4899300 (3404485, 6533347)	115	917 (649, 1222)	862 (605, 1153)	-6
**Neck pain**	Males	283699 (185575, 415015)	702614 (457845, 1029227)	148	237 (156, 343)	236 (155, 344)	0
	Females	424158 (279732, 619412)	1026514 (671847, 1507591)	142	373 (248, 546)	375 (246, 547)	0
	Both	707857 (466302, 1024804)	1729128 (1136538, 2521212)	144	304 (202, 440)	303 (202, 439)	0
**Other MSK**	Males	233236 (153401, 332732)	876821 (585692, 1252590)	276	190 (126, 270)	289 (193, 410)	52
	Females	443858 (308221, 613643)	1420682 (983884, 1973366)	220	379 (263, 521)	513 (358, 703)	35
	Both	677093 (464532, 941330)	2297502 (1568891, 3212578)	239	282 (194, 394)	397 (270, 549)	41

**Fig. 3:**
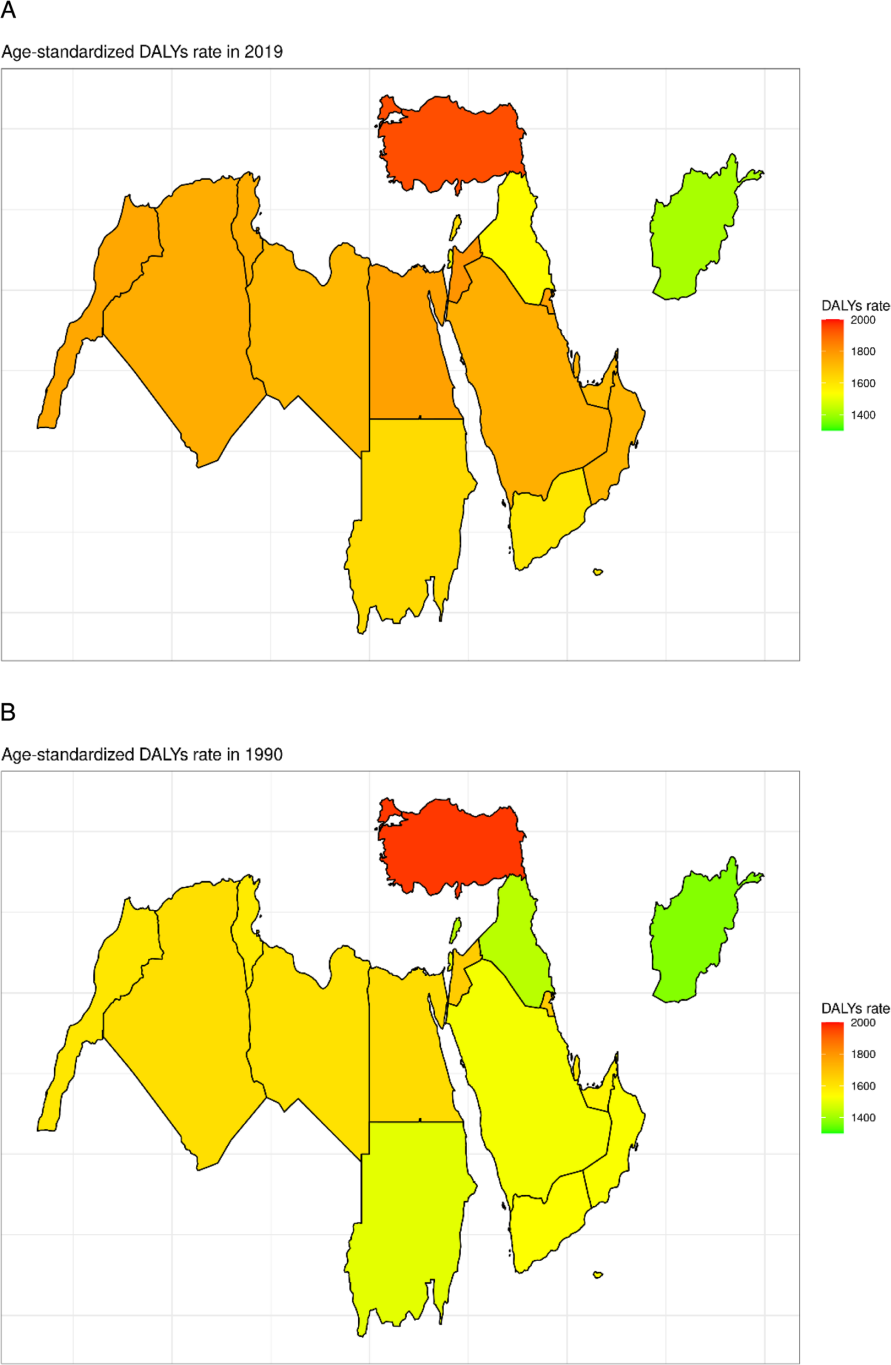
**A** Age-standardized DALYs across MENA region countries in 2019. **B** Age-standardized DALYs across MENA region countries in 1990

**Fig. 4:**
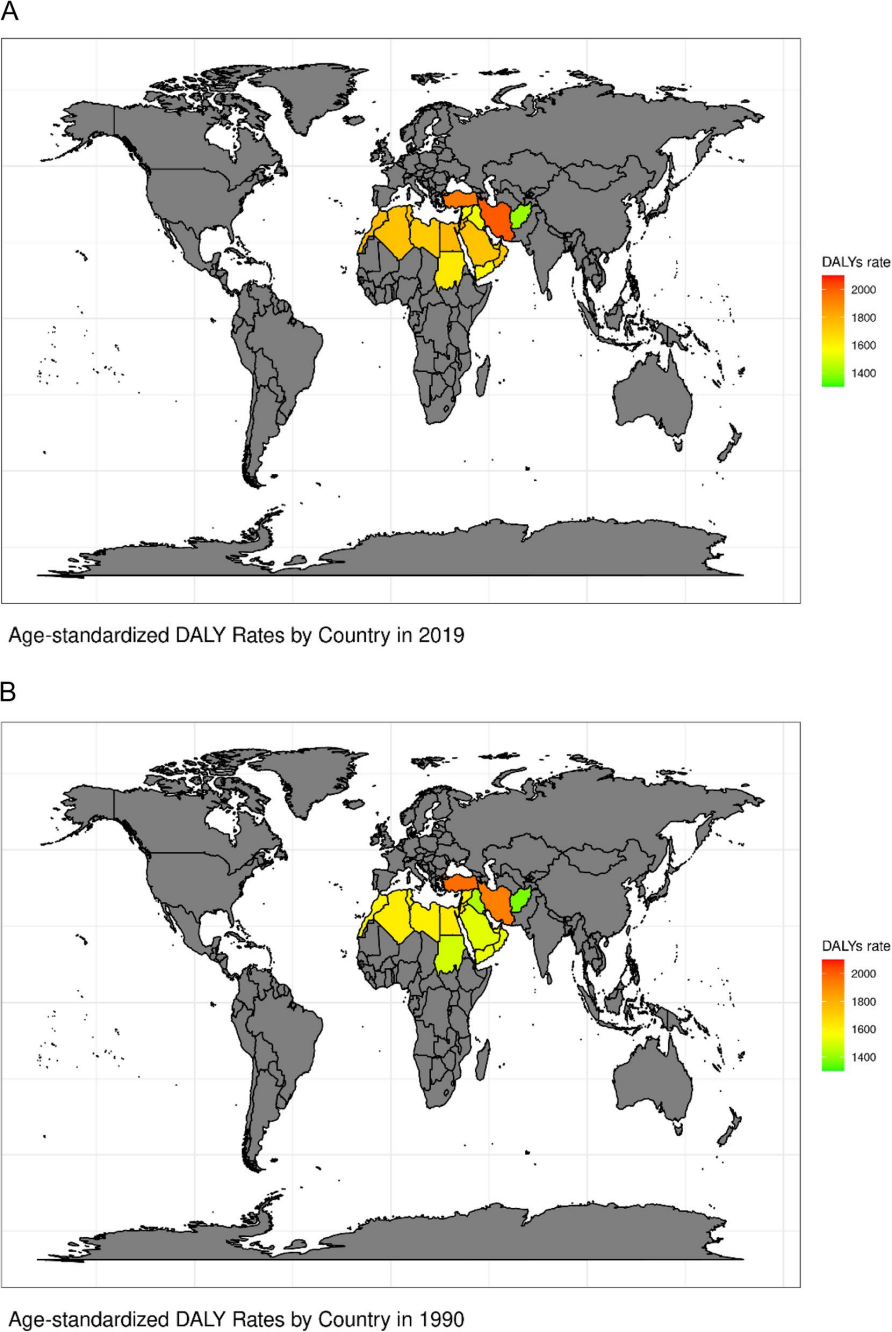
**A** Age-standardized DALYs across MENA region countries in 2019, mapped to show MENA regions in a global context. **B** Age-standardized DALYs across MENA region countries in 1990, mapped to show MENA regions in a global context

In terms of national variation across the region, Afghanistan had the lowest age standardized DALYs rate attributed to MSK disorders (1408 per 100,000; 95% UI = 980 – 1899), followed by Palestine (1540 per 100,000; 95% UI = 1065 – 2042) and Iraq (100,000 1540 – 2065). On the other hand, the three countries with the highest age standardized DALYs due to MSK disorders were Iran, Turkey, and Jordan, with rates of 2007 (95% UI = 1440 – 2655), 1938 (95% UI = 1402 – 2565), and 1806 (95% UI = 1303 – 2379), respectively. As for the changes in age standardized DALY rates from 1990 to 2019, Syria/Syrian Arab Republic exhibited the most dramatic decrease from 1618 per 100,000 to 1549 per 100,000, while Saudi Arabia had the most notable increase from 1518 per 100,000 to 1742 per 100,000. National variations in DALYs rates in 1990 and 2019 are shown in Fig. 3A and B. Additionally, Fig. 4A and B show the age-standardized DALYs across MENA region countries in 2019 and 1990, respectively, mapped to show MENA regions in a global context.

#### Burden of MSK disorders by age

**Fig. 5:**
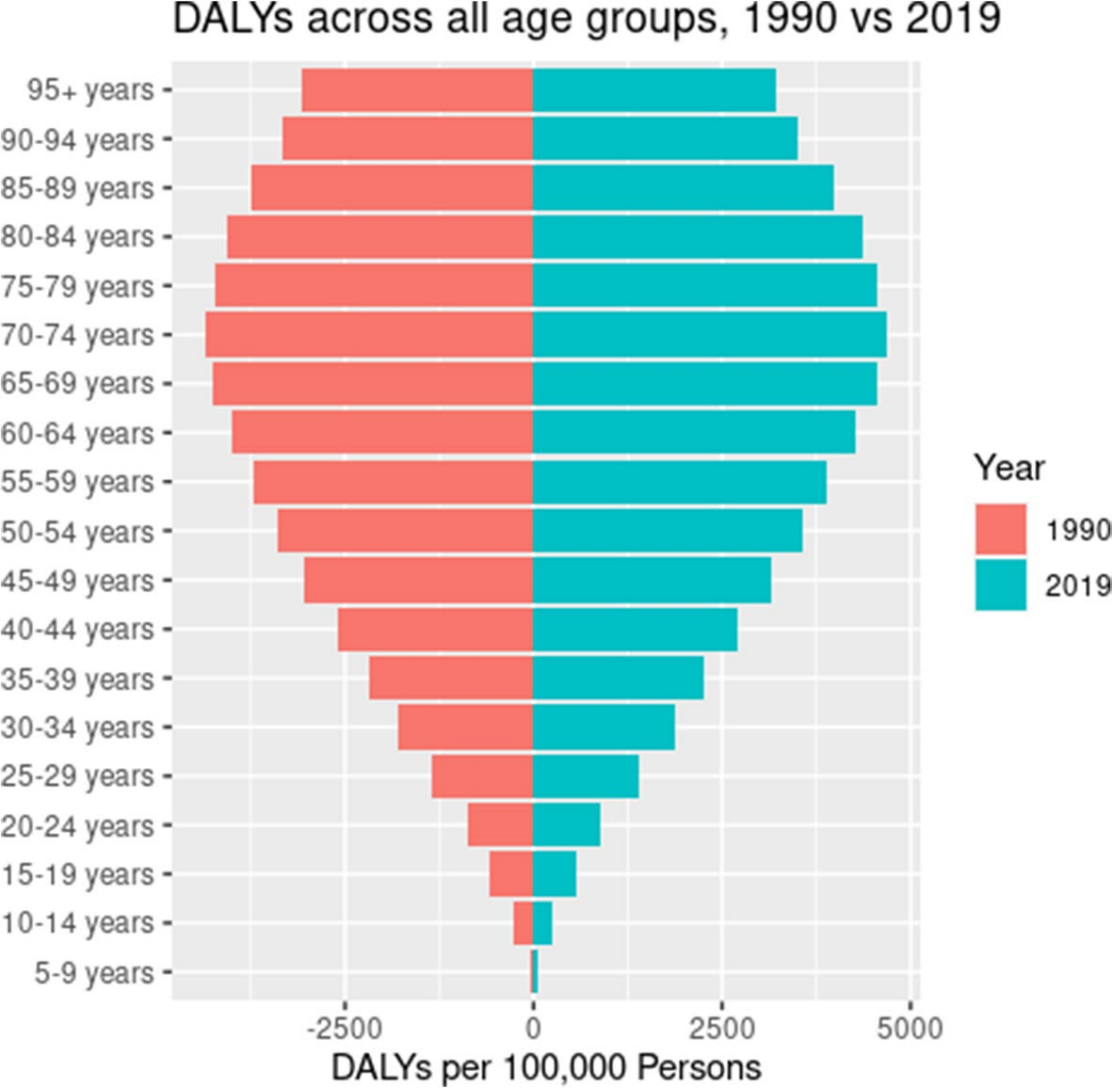
DALYs for MSK disorders in 1990 and 2019, by age groups

The burden of MSK disorders reached its peak in the 70 – 74 years old age group in 2019. This was similar to the pattern observed in 1990, and there was no shift in age dynamics between 1990 – 2019 (Fig. 5). Between 1990 and 2019, the 75 – 79 years old age group witnessed the largest increase in burden of MSK disorders (DALYs age-standardized rate increased by 343). Similarly, the age group that witnessed the least change in burden was those aged 15 – 19 years old (DALYs age-standardized rate decreased by 10.4). Other age groups that had a decrease in the burden of MSK disorders were pediatric age groups (e.g., 5—9 years old and 10 – 14 years old).

### Risk Factors for MSK disorders

**Fig. 6:**
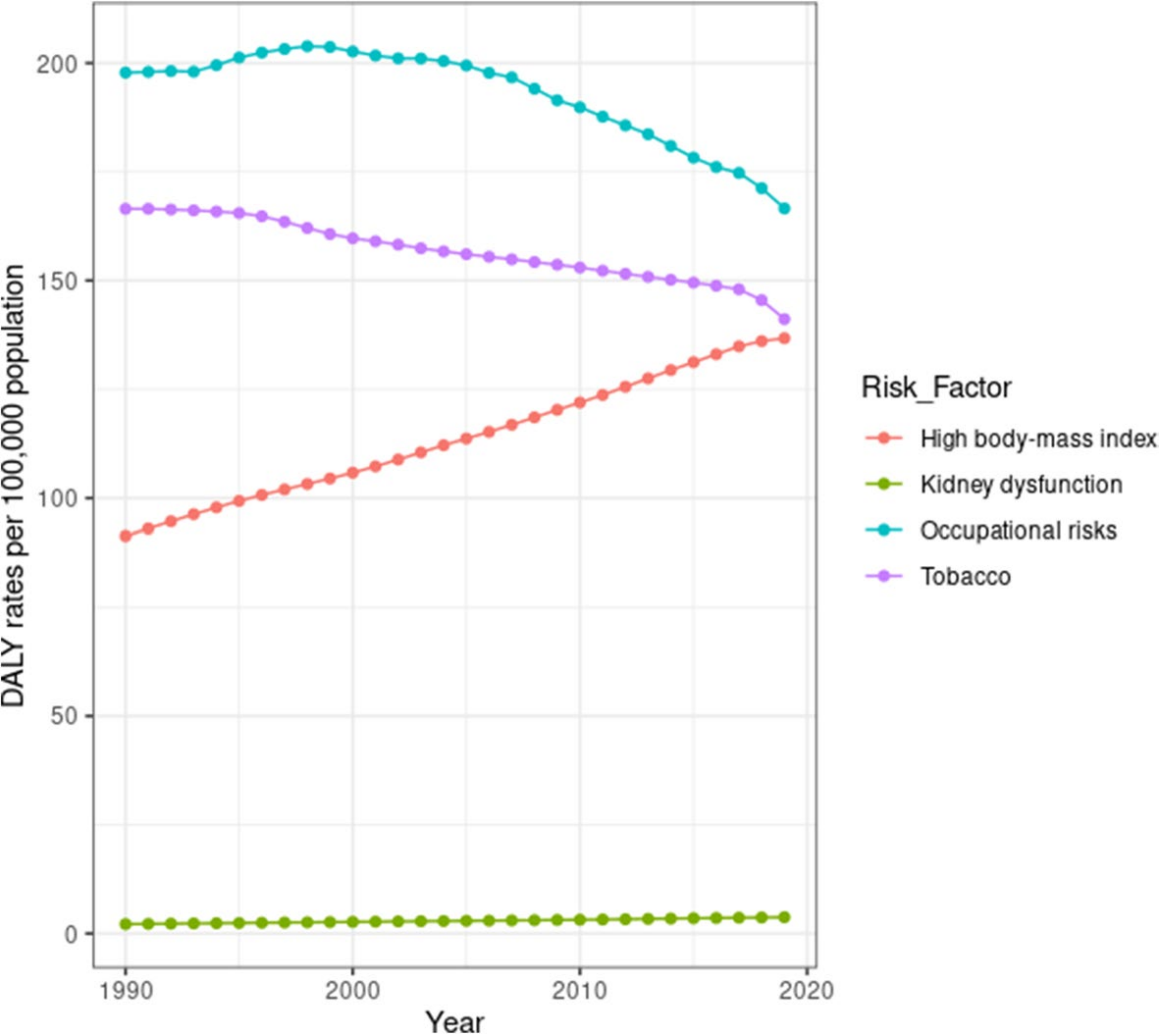
Contribution of main risk factors to MSK disorders DALYs from 1990 to 2019

**Fig. 7:**
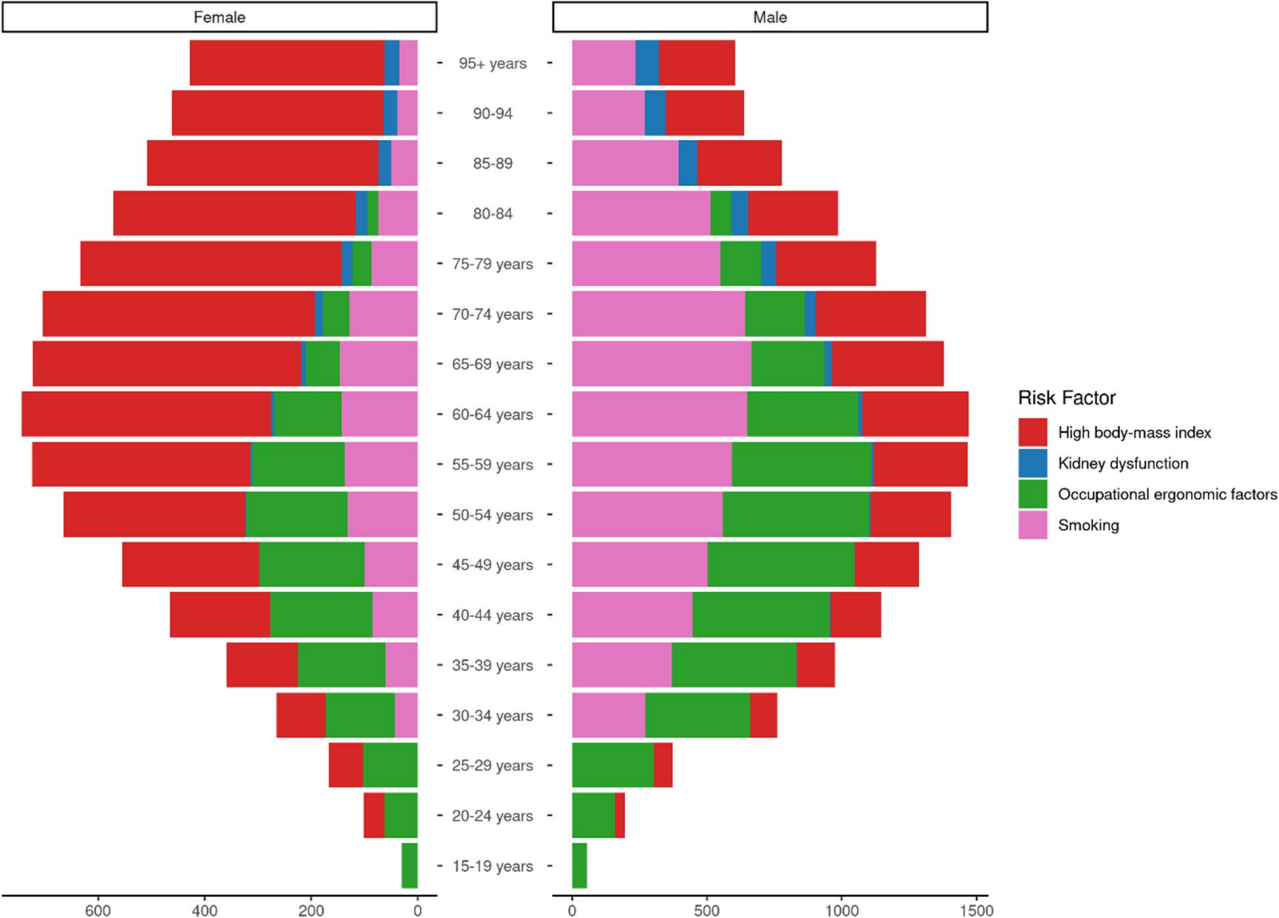
Contribution of main risk factors to MSK disorders DALYs in 2019, stratified by gender and age groups

In 2019, occupational risks, tobacco smoking, and high body mass index were the main risk factors for DALYs of MSK disorders, accounting for 166 per 100,000 (95% UI = 113 – 227), 137 per 100,000 (95% UI = 75 – 221), and 166 (95% UI = 104 – 237), respectively. Additionally, kidney dysfunction was a minor risk factor, contributing merely to 3.74 per 100,000 age standardized DALYs for MSK disorders (95% UI = 2 = 5). Between 1990 and 2019, there was a change in the trend of highest risk factors contributing to MSK disorders. Between 1990 and 2019, occupational risks age-standardized DALYs contribution decreased from 198 per 100,000 (95% UI = 134 – 266) to 166 per 100,000 (95% UI = 113 – 227). Despite this, occupational risks remained the largest risk factor for MSK disorders burden. On the other hand, the contribution of high body mass index as a risk factor for MSK disorders burden increased linearly from 91 per 100,000 age-standardized DALYs (95% UI = 45 – 158) to 137 per 100,000 (95% UI = 75 = 221). Figure 6 demonstrates the change in age standardized DALYs for each of the four risk factors between 1990 and 2019. Finally, risk factor contribution to standardized DALYs differed according to gender and age groups. Among females, high body mass index was the highest contributing risk factor, while smoking was for males Fig. 7. Contribution of main risk factors to MSK disorders DALYs in 2019, stratified by gender and age groups.

## Discussion

The objective of this study was to evaluate the burden of MSK disorders in the MENA region, as well as their relevant risk factors, from 1990 to 2019. To the best of our knowledge, this is the first study to thoroughly report the burden of MSK disorders among a very understudied region in health research. Overall, MSK disorders are serious and carry a high burden of disability and should be prioritized by health policy makers to achieve better health outcomes and life quality measures among populations. In this study, we reported the burden of MSK disorders in the MENA region and highlighted national variations in the burden, demonstrating that Iran, Turkey, and Jordan are the three countries with highest burden of MSK disorders in the MENA region. To date, this study provides the first national-level comparisons regarding this topic among different MENA countries. Such findings can inform local health systems to prioritize MSK disorders and employ various preventative and community-level interventions to address the rising burden of MSK disorders.

To best direct intervention measures and formulate policies that address the burden of MSK disorders among populations, research on risk factors contributing to MSK disorders is necessary. Our findings revealed that occupational risk contributed the most to MSK disorders. This is in line with other findings in the literature regarding the biggest risk factors to MSK disorders globally [19]. In the past 30 years, this has shifted dramatically, and the risk associated with occupational hazards has decreased in their contribution to MSK disorders, arguably due to a shift in the nature of work with the rapid technological advancement witnessed. This includes a shift from manual labor work and farming. In hands-on occupations (e.g., farming, mechanic) that require physical stress, prolonged standing and static postures can increase spinal load and spinal contraction. In turn, this leads to changes in biomechanics, physiology, and neurological function that may heavily contribute to the prevalence of different MSK disorders [35]. Furthermore, it is important to note that high BMI increased its contribution to MSK disorders drastically from 1990 to 2019, and constitutes a big risk factor for Osteoarthritis, low back pain, and other musculoskeletal disorders. This highlights the need for health education interventions that can target common causes such as diet, alcohol consumption, physical inactivity and obesity [36]. Additionally, intervention measures should address tobacco smoking given its contribution to low back pain, rheumatoid arthritis, and other musculoskeletal disorders. Tobacco smoking may be associated with increased musculoskeletal disorders as it causes general damage to musculoskeletal tissues through vasoconstriction, hypoxia, defective fibrinolysis, or other mechanisms that impair their nutrition or structure [37–39]. Moreover, policies promoting active lifestyle and healthy diets can potentially improve the burden of MSK disorders in the region. Social and physical environmental factors are both associated with increased physical activity among the population. This includes promoting social cohesion [40–42], enabling access to appropriate exercise facilities [43], and utilizing urban design to increase neighborhood walkability [44–46], and green spaces [47–49].

In addition to micro-level interventions focusing on educating the general populations, the findings of this paper illustrate the necessity for an interdisciplinary and cross-sector approach to screen for and treat MSK disorders in the region adequately and effectively [50]. While healthcare systems differ from one country to another in the region, most health systems prioritize curative health care actions, particularly in the context of low-income and crisis settings. Additionally, health systems and policies in the region tend to be less responsive to conditions that are less frequently associated with mortality, such as MSK disorders. In turn, these conditions are less emphasized and prioritized when it comes to the development of policies and clinical guidelines to address them [51, 52]. With the increasing burden for MSK disorders, there is a strong call to shift healthcare systems priorities to foster more integrative care that considers promotion, prevention, and rehabilitation.

Designing tailored interventions and public health policies to address risk factors differently among gender and different age groups in the MENA region is crucial to ensure effective prevention and management of MSK disorders. Our findings demonstrate that risk factors for MSK disorders vary significantly among different population groups. For instance, smoking is the most significant risk factor for MSK disorders among males, while high BMI is a greater risk factor for females. Moreover, for pediatric patients and young adults, occupational ergonomic factors were the biggest risk factors for MSK disorders. Therefore, a "one-size-fits-all" approach may not be appropriate, and policies need to be tailored to address these differences in risk factors. By designing targeted interventions and public health policies that account for gender and age group differences, we can help prevent and manage MSK disorders more effectively and promote better health outcomes for individuals in the MENA region.

### Strengths and limitations

This study utilized the latest available data from the GBD project. The 2019 version of GBD has seen several major changes compared to previous versions. Firstly, the 2019 dataset has expanded the number of causes and risks included, resulting in a more comprehensive analysis of the global burden of disease. Secondly, there have been improvements in data quality and availability, especially in low- and middle-income countries, which has led to a more accurate representation of disease burden. Thirdly, the methodology used in GBD 2019 has been updated to reflect new scientific evidence, such as incorporating new disease classifications and refining estimates of uncertainty [25]. In the context of the MENA region, the GBD 2019 dataset provides the most comprehensive population-level data regarding health outcomes. Furthermore, this study adds to the literature by thoroughly exploring a topic that is very understudied in epidemiological research and provides evidence-based directions for intervention measures and health policies. Additionally, public health data continues to be lacking in the MENA region, and with the absence of any efforts to increase the presence of rehabilitation medicine as a specialty in the region, our study provides the first quantifiable measures to present a strong call of action. Finally, this study analyzed the risk factors for MSK disorders, which can be beneficial for prevention measures on the population level.

Despite those strengths, this study is not without noteworthy limitations. The main limitation of this study is its reliance on secondary data. Despite the GBD's great efforts to provide reliable data using advanced modeling and estimation methods based on primary data, data derived from nations can be inaccurate and patchy [53]. In certain Middle Eastern countries, data collection systems may be well-established and integrated with healthcare facilities, providing accurate and comprehensive data on causes of death. However, in other Middle Eastern nations, data collection may not be as well-established, resulting in significant disparities in data quality and availability across different countries, particularly in remote and rural areas. Overall, the estimates presented are subject to potential errors and biases due to limitations in the quality, completeness, and availability of data, which may have resulted in under or overestimation of the true burden of MSK disorders in the region. Additionally, data may be subject to change due to changes in disease classification or because of continued social unrest and rapid immigration within the region [54]. Moreover, some of the other conditions of MSK disorders were classified under one category (e.g., other MSK disorders) and hence we were unable to evaluate them separately [19]. Another limitation was the lack of distinction between acute and chronic low back pain, which can be considered separate entities with different etiologies clinically. This would have provided more precise findings and offer more specialized intervention and policy directions. Furthermore, it is important to acknowledge that the data only demonstrates the association of risk factors and MSK disorders. Hence, this cannot imply causation and the findings of this paper must be interpreted accordingly. As an ecological study, our findings may be subject to ecological fallacy and the lack of individual-level data limits the ability to draw conclusions at the individual level. Finally, we acknowledge that there may be important confounders and variables that could interact with DALYs over time, including lifestyle factors, comorbidities, access to healthcare, and treatment modalities. However, this limitation is inherent to the GBD study and was non-modifiable but must be acknowledged when interpreting the results.

### Future research

Future research should continue to investigate the burden of MSK disorders in the MENA region and beyond, utilizing other datasets and sources to validate the current findings and provide extensive data on the topic. Additionally, future research should attempt to employ other study designs, including longitudinal studies to establish causation between different risk factors and MSK disorders. Moreover, understanding the impact of other variables on the burden of MSK disorders can be beneficial, including socioeconomic status and other genetic variables.

## Conclusion

MSK disorders carry a large burden of disease in the MENA region, and multiple risk factors contribute to this increased burden in the past decades. This includes occupational risks, tobacco smoking, high BMI, and kidney dysfunction. Interventions that address risk factors have the potential to improve health outcomes among the population. Future research should continue to explore the burden of MSK disorders and better understand how to intervene, especially among an understudied population in public health research such as that of the MENA region.

## Data Availability

All data used in this study are freely available online at: http://ghdx.healthdata.org/gbd-results-tool.
